# Vitamin D alleviates non-alcoholic fatty liver disease *via* restoring gut microbiota and metabolism

**DOI:** 10.3389/fmicb.2023.1117644

**Published:** 2023-02-02

**Authors:** Xiao-Lei Zhang, Lei Chen, Jiang Yang, Shan-Shan Zhao, Shi Jin, Na Ao, Jing Yang, Hui-Xin Liu, Jian Du

**Affiliations:** ^1^Department of Endocrinology, The Fourth Affiliated Hospital of China Medical University, Shenyang, Liaoning, China; ^2^Institute of Health Sciences, China Medical University, Shenyang, Liaoning, China; ^3^Institute of Life Sciences, China Medical University, Shenyang, Liaoning, China; ^4^Liaoning Key Laboratory of Obesity and Glucose/Lipid Associated Metabolic Diseases, China Medical University, Shenyang, Liaoning, China

**Keywords:** gut microbiota, NAFLD, vitamin D, sphingolipid metabolism, tyrosine metabolism

## Abstract

**Background:**

Non-alcoholic fatty liver disease (NAFLD) represents a severe public health problem. Dysbiosis of gut microbiome has been identified as one of the key environmental factors contributing to NAFLD. As an essential nutrition, Vitamin D (VD) plays an important role in regulating gut microbiota based on its receptor (Vitamin D Receptor, VDR) which is highly expressed in the gastrointestinal tract.

**Methods:**

Rats were fed with HFD (high-fat diet) for 12 weeks. And the rats were treated with VD two times a week by intraperitoneal injection for 12 weeks. H&E staining combined with plasma biochemical index was performed to characterize pathological changes and function of the liver. Fecal microbiota 16S rRNA gene sequencing and metabolomics were taken to reveal the altered gut microbiota and metabolites.

**Result:**

The VD alleviates the HFD-induced lipid accumulation in the liver as well as decreases the levels of amlodipine besylate (ALT) and amlodipine aspartate (AST). VD supplement decreased the ratio of phylum Firmicutes/Bacteroidetes (F/B) but increased alpha diversity. In addition, the VD treatment improved the HFD-induced gut microbiota by increasing the Prevotella and Porphyromonadaceae and decreasing Mucispirillum, Acetatifactor, Desulfovibrio, and Oscillospira abundance. Furthermore, the capability of tyrosine metabolism, tryptophan metabolism, arginine biosynthesis, and sphingolipid metabolism was enhanced after VD treatment. Consistently, Prevotella positively correlated with tryptophan metabolism and sphingolipid metabolism. Importantly, the Prevotella abundance was positively associated with serotonin, melatonin, tryptamine, L-arginine, and 3-dehydrosphinganine which synthesize from tryptophan, tyrosine, arginosuccinate, and serine, respectively.

**Conclusion:**

VD treatment inhibited HFD-induced NAFLD accompany by dysbiosis gut microbiota and metabolites, suggesting that VD supplement could be a potential intervention used for NAFLD treatment by targeting the specific microbiota.

## Introduction

The gut microbiome has been identified as an essential mediator in the occurrence and development of non-alcoholic fatty liver disease (NAFLD), the most prevalent chronic liver disease, by regulating host energy and metabolism (Brunt et al., 2015; Sookoian et al., 2020). Human cross-sectional studies show an increased ratio of phylum *Firmicutes*/*Bacteroidetes* (F/B) and a decreased butyrate-producing bacteria Ruminococcaceae involved in NAFLD progression (Mouzaki et al., 2013). The clinical study discovered that the *Proteobacteria* phylum was enriched in patients with NAFLD (Aron-Wisnewsky et al., 2020a). Gut microbiota may modulate NAFLD through carbohydrate absorption and nutrient metabolism as well as producing short-chain fatty acids (SCFAs), which link the gut microbiota and physiology, especially hepatic gluconeogenesis. For example, gut microbiota *Enterococcus* metabolize trimethyllysine (TML) to N,N,N-trimethyl-5-aminovaleric acid (TMAVA), which could aggravate fatty liver (Zhao et al., 2020). Also, the gut microbiota metabolite indole alleviates diet-induced NAFLD *via* resisting inflammatory responses (Ma et al., 2020). In addition, fecal microbiota transplantation (FMT) from high-fat diet-fed mice accelerates steatosis and impairs insulin (Bauer et al., 2022). Furthermore, FMT could decrease fat accumulation in the liver by improving gut microbiota dysbiosis, thus attenuating fatty liver disease (Aron-Wisnewsky et al., 2020b). A previous study revealed that *Lactobacillus* supplement-driven reprogramming of gut microbiome and metabolome ameliorates the progression of NAFLD (Yu et al., 2021). Thus, the gut microbiota might be the novel therapeutic concept for counteracting the development of NAFLD.

Vitamin D (VD) and its active form 1,25-dihydroxyvitamin D (1,25D) inhibit immune responses and indirectly attenuate immune responses by increasing IL-10 production in macrophages, dendritic cells, and T cells (Cantorna et al., 2019). Our previous study showed that VD supplement may attenuate diet-induced liver injury by inhibiting pyroptosis (Zhang et al., 2021c). Other mechanisms underlying VD therapy against NAFLD should be considered. Furthermore, the serum 1,25D level is correlated with the α-diversity of gut microbiota and butyrate-producing bacteria, leading to better gut microbial health (Thomas et al., 2020). Importantly, the vitamin D receptor (VDR) is highly expressed in the gastrointestinal tract where it regulates gene expression (Thomas et al., 2020). A significant shift in the microbiota and serum measurements of selected bile acids and fatty acids was discovered in *Vdr* knockout mice compared to control mice (Wang et al., 2016). Thus, a VD supplement might alleviate NAFLD by restoring the dysbiosis gut microbiota and metabolism.

Here, the high-fat diet (HFD)-induced NAFLD rat model was adopted to treat VD by intraperitoneal injection. VD supplement obviously reversed the serum transaminases, hepatic fat deposits, and hepatocyte membrane destruction induced by HFD. In addition, VD decreased plasma glucose profiles. Furthermore, VD treatments stimulated the growth of *Porphyromonadaceae* and *Prevotella* but decreased *Mucispirillum*, *Acetatifactor*, *Desulfovibrio*, and *Oscillospira*. The tyrosine metabolism, tryptophan metabolism, arginine biosynthesis, and sphingolipid metabolism were enhanced by VD treatments. Of note, sphingolipid metabolism was positively correlated with *Prevotella*. Taken together, targeting gut microbiota and the sphingolipid metabolism pathway may be a feasible preventive strategy for patients with NAFLD.

## Materials and methods

### Animals and diets

Five-week-old male SD rats were housed in a specific pathogen-free environment at 20–22°C with 12-h light–dark cycles. To establish the NAFLD model, rats were fed with an HFD (HFD group, protein, 20%; fat 60%, carbohydrates, 20%) after 1 week of adaptation for 12 weeks. Normal diet combined with VD supplement (ND + VD) and HFD with VD supplement (HFD + VD) groups’ rats were treated with 1,25(OH)_2_D_3_ (5 μg/kg, Cayman, USA) two times a week by intraperitoneal injection for 12 weeks. Rats in ND and HFD groups were intraperitoneally injected with an equivalent volume of vehicle. During the experiments, body weight and food intake were assayed weekly. All animal procedures used in this study were conducted according to the Guide for Care and Use of Laboratory Animals and approved by the ethics committee of China Medical University (No. 2018162). At the end of the experiment, animals were euthanized to harvest liver, blood, and feces for further analysis.

### Biochemical analysis

Plasma glucose, amlodipine besylate (ALT), amlodipine aspartate (AST), triglyceride (TG), and total cholesterol (TC) levels were quantified according to the manufacturer’s instructions (Nanjing Jiancheng, China).

### Histological and microscopy analyses

Histological changes in liver tissues were detected using hematoxylin–eosin (H&E) staining. The fixed liver tissues were embedded in paraffin and cut into 5-μm thick slices.

The liver sections were analyzed by transmission electron microscopy.

### Fecal microbiota 16S rRNA gene sequencing

Stool DNA was isolated, and the V3 + V4 hypervariable regions of the bacterial 16S rRNA gene were amplified using primers CCTACGGGNGGCWGCAG(F) and GACTACNVGGGTATCTAAT(R). After PCR products were purified, the positive amplicon sequencing was performed on the Illumina MiSeq PE300 platform. After cutting off barcodes, QIIME V.2.0. was used for analyzing raw sequencing reads (Caporaso et al., 2010; Yang et al., 2022). Uparse software (version 7.1) was used to cluster the same operational taxonomic units (OTUs) with ≥97% similarity sequences. The Greengenes 16S rRNA gene reference database was adopted to classify OTU taxonomically.

### Analysis of flora microbiota diversity, structure, and predictive function

The OTU numbers of each sample were flattened, and the alpha diversity of fecal bacteria was calculated based on the normalized OTU table by R package Vegan as previous methods (Sheng et al., 2017; Wang et al., 2018; Zhang et al., 2022). Principal coordinate analysis (PCoA) and similarities (PERMANOVA) were used to reveal the difference in stool microbiome profile based on the OTU level, which was analyzed by R package Vegan (Liu et al., 2022; Zhao et al., 2022). In addition, the sequence of OTUs in samples was used to predict the function of the intestinal microbiome by PICRUST2 as previously described (Hu et al., 2015; Liu et al., 2016), and significantly changed KEGG pathways were tested by Kruskal–Wallis and *post-hoc* Dunn’s test.

### Analysis of plasma metabolomics

Blood metabolite signatures were identified by LC-MS between HFD and HFD + VD groups samples. The processed data such as m/z, RT, and normalized peak area percentages were imported into SIMCA to identify metabolites. The HMDB database was adopted to map and identify the metabolites. Partial least squares discriminant analysis (PLS-DA) was used to reveal the metabolite changes in groups by R package ropls, and the abundance of significant metabolites with variable important in projection (VIP) ≥1 and *p*-value (Wilcoxon’s test) < 0.05 was selected for enrichment analysis. The enrichment pathway of differential plasma metabolite profile between the two groups was analyzed by MetaboAnalyst 5.0, respectively.^1^

### Correlation and co-occurrence network analysis

The relationship of KEGG pathways of bacterial predicted function and remarkable changed bacterial, alpha diversity of microbiota and microbiome as well as blood metabolites and gut bacterial were analyzed by Spearman’s correlation and Mantel’s test based on R package dplyr and ggcor. Meanwhile, the co-occurrence network of metabolites of key pathways and changed bacteria was constructed and visualized by R package igraph and Cytoscape, respectively.

### Statistical analysis

For statistics in multiple groups, we utilized Kruskal–Wallis and two-way ANOVA tests to evaluate the difference among groups, respectively. *Post-hoc* Dunn’s test, Tukey’s multiple-comparison test, and Wilcoxon’s test were performed to analyze the difference between the two groups. *P*-values < 0.05 were considered statistically significant. Error bars indicate mean ± standard error of mean (SEM).

## Results

### VD intake alleviated the HFD-induced NAFLD features and liver injury

**FIGURE 1:**
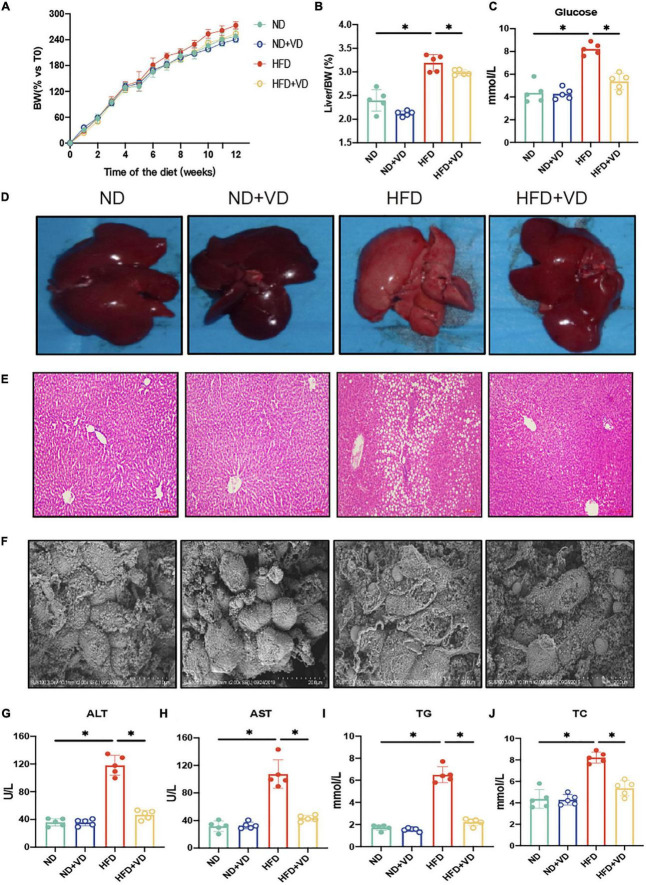
Vitamin D (VD) supplement alleviates high-fat diet (HFD)-induced non-alcoholic fatty liver. **(A)** The body weight (BW) of male rats fed indicated diet was measured weekly for 12 weeks and expressed as percentage vs. time 0. Data are mean values ± SEM (*n* = 5). **(B,C)** The liver organ index and fasting blood glucose in four groups. **(D–F)** Representative gross liver morphology, hematoxylin–eosin (H&E)-stained liver sections, and electron microscope picture of the liver. **(G–J)** The plasma amlodipine besylate (ALT), amlodipine aspartate (AST), total cholesterol (TC), and triglyceride (TG) levels in indicated groups. *P < 0.05. *P*-values are derived from ordinary two-way ANOVA followed by Tukey’s multiple-comparison test.

As shown in Figures 1A–C, the HFD remarkably increased body weight (BW), liver/BW ratio, and fasting serum glucose more than ND. The VD treatment significantly decreased the aforementioned index relative to HFD-fed rats. In addition, progressive lipid droplet accumulation was observed in the livers of the HFD group *via* H&E staining (Figures 1D–E). Furthermore, the VD could inhibit hepatocyte swelling and membrane rupturing compared to the livers of the HFD group, as revealed by electron microscopy (Figure 1F). Consistent with the histological changes, serum ALT, AST, TG, and TC were also significantly decreased after VD intervention compared to those of the HFD-fed rats (Figures 1G–J).

### Alterations of gut microbiota upon VD treatments in NAFLD rats based on the 16S rRNA gene

**FIGURE 2:**
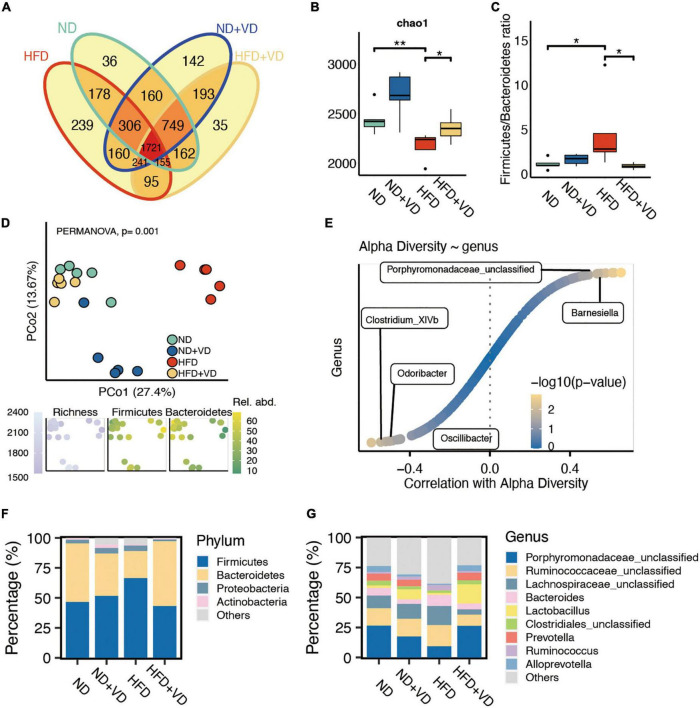
Altered fecal microbiota after vitamin D (VD) treatment. **(A)** The shared and unique observed operational taxonomic units (OTUs) are in four groups. **(B)** The alpha diversity difference among groups. **(C)** The comparison of phylum Firmicutes/Bacteroidetes ratio in four group samples. **(D)** Principal coordinate analysis (PCoA) reveals a clear distinct difference in gut microbiota in four groups. **(E)** The correlation between alpha diversity and alerted gut microbiome. **(F,G)** Stacked bar plots depicting the percentage of phylum (left) and genus (right) of intestinal bacteria in the screening results. *P < 0.05, **P < 0.01. Kruskal–Wallis test with *post-hoc* Dunn’s test.

As shown in Figure 2A, the shared OTUs between HFD + VD and ND (2,787 = 1,721 + 155 + 749 + 162) were more than that between HFD + VD and HFD (2,212 = 1,721 + 155 + 241 + 95). Furthermore, the chao1 index in the HFD group significantly decreased compared to that in the ND group (Figure 2B), but the increased chao1 index was observed in the rats after VD treatment. We also found that VD intervention could significantly decrease the phylum *Firmicutes*/*Bacteroides* ratio which was induced by HFD (Figure 2C). In addition, the PCoA analysis revealed clear distinct discrimination in four groups (Figure 2D). Specifically, there was a closer distance between HFD + VD and ND rats than that between HFD + VD and HFD (Figure 2D), and the richness index was driven by the F/B ratio (Figure 2D). Moreover, *Barnesiella* and *Porphyromonadaceae_unclassified* genus were positively correlated with alpha diversity (Figure 2E). The negative association between alpha diversity and *Clostridium*, *Odoribacter*, and *Oscillibacter* genera was also revealed by Spearman’s correlation analysis (Figure 2E). It was noted that the VD treatment increased the proportion of phylum *Bacteroidetes* compared to HFD-fed rats (Figure 2F). The decrease in phylum *Firmicutes* was also observed in rats of HFD + VD relative to that in the HFD group (Figure 2F). At the genus level, the VD supplement reversed the decreased abundance of *Porphyromonadaceae_unclassified* and *Prevotella* genus, which only showed in the HFD group (Figure 2G). More importantly, the VD supplement could also boost the abundance of *Lactobacillus* genus even feeding with HFD (Figure 2G).

### The specific change in gut microbiota after VD intervention in NAFLD rats

**FIGURE 3:**
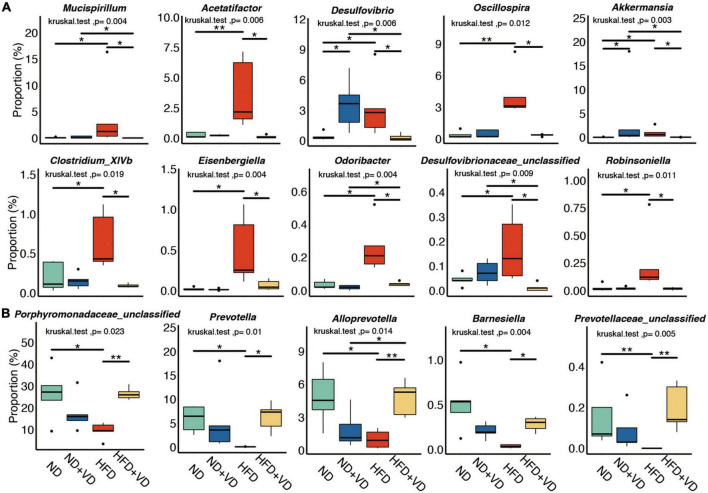
Identification of specific altered bacteria for vitamin D (VD) intervention. **(A,B)** Significantly changed gut microbiome in four groups. *P < 0.05, **P < 0.01. Kruskal–Wallis test with *post-hoc* Dunn’s test.

Next, we compared the species level change after VD treatment. As shown in Figure 3A, HFD significantly increased the abundance of *Mucispirillum, Acetatifactor, Desulfovibrio, Oscillospira, Clostridium_XlVb, Eisenbergiella, Odoribacter*, and *Robinsoniella* species, but the aforementioned bacteria were all downregulated after VD treatment. However, the supplement of VD reversed the decreased proportion of *Porphyromonadaceae_unclassified, Prevotella, Alloprevotella*, and *Barnesiella* which were induced by HFD (Figure 3B).

### VD altered the microbial function and metabolic pathway

**FIGURE 4:**
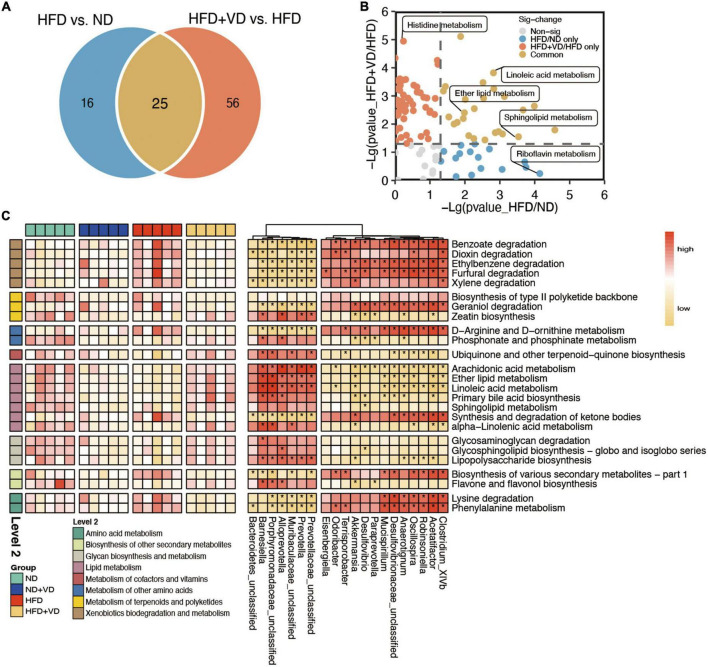
The characteristic function of gut microbiota. **(A)** Venn diagrams illustrating the number of predicted pathways significantly changed between high-fat diet (HFD) vs. ND and HFD + VD vs. HFD. **(B)** The clear distinct difference between HFD vs. ND and HFD + VD compared to HFD, respectively. **(C)** The predicted function of bacteria and the connection between the predicated function of intestinal microbiota and altered gut microbiota. Red denotes positive correlations. Yellow denotes negative correlations. *P < 0.01.

Furthermore, the predicted function of gut microbiota revealed that there was a clear distinct change in metabolism pathways. As shown in Figure 4A, apart from 25 pathways overlapping between HFD compared to ND and HFD + VD, the VD treatment uniquely changed the 56 pathways relative to HFD. Specifically, linoleic acid metabolism, ether lipid metabolism, and sphingolipid metabolism pathways were enriched in both two comparisons (Figure 4B), and the histidine metabolism and riboflavin metabolism were uniquely changed after VD and HFD supplement, respectively (Figure 4B). Importantly, arachidonic acid metabolism, linoleic acid metabolism, ether lipid metabolism, and sphingolipid metabolism which belong to lipid metabolism were increased after VD treatment (Figure 4C). Decreased amino acid metabolism, containing lysine degradation and phenylalanine metabolism, was downregulated upon VD supplement. Finally, the consisting higher levels of *Porphyromonadaceae_unclassified, Prevotella, Alloprevotella*, and *Barnesiella* after VD treatment were positively correlated with lipid metabolism, which negatively associated with *Mucispirillum, Acetatifactor*, and *Desulfovibrio* abundance (Figure 4C).

### Metabolomics revealed the VD intake altered specific metabolic pathways

**FIGURE 5:**
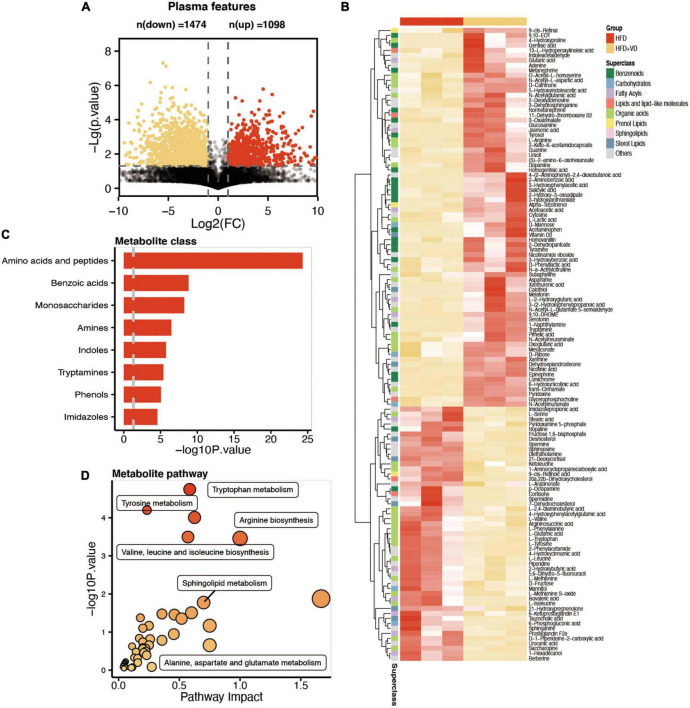
Comparative metabolomics analysis determines the change in plasma metabolites in high-fat diet (HFD) + VD from HFD. **(A)** The volcano plot shows the number of dysregulated metabolite features between the two groups. **(B)** The heatmap shows the annotated changed metabolites in the plasma profile. **(C,D)** The metabolic set and pathway enrichment based on the different metabolites.

Plasma samples from the samples were analyzed by the global metabolite panel, which identified a significant change in 1,098 (up) and 1,474 (down) features after VD treatment (Figure 5A). As shown in Figure 5B, the L-valine, L-leucine, and L-isoleucine were significantly decreased after VD treatment. Also, the sphingosine, L-serine, and sphinganine were decreased in the HFD + VD group, but a higher level of 3-dehydrosphinganine, vitamin D3, L-arginine, and dopamine was observed after VD intervention. The metabolic set analysis revealed that the changed metabolites are mainly enriched in amino acids, peptides, benzoic acids, monosaccharides, amines, and indoles (Figure 5C). The changed metabolites are shown in Supplementary Table 1. Furthermore, the KEGG pathway enrichment indicated tyrosine metabolism, tryptophan metabolism, arginine biosynthesis, valine, leucine, and isoleucine biosynthesis, and sphingolipid metabolism pathways were remarkably altered after VVD treatment (Figure 5D).

**FIGURE 6:**
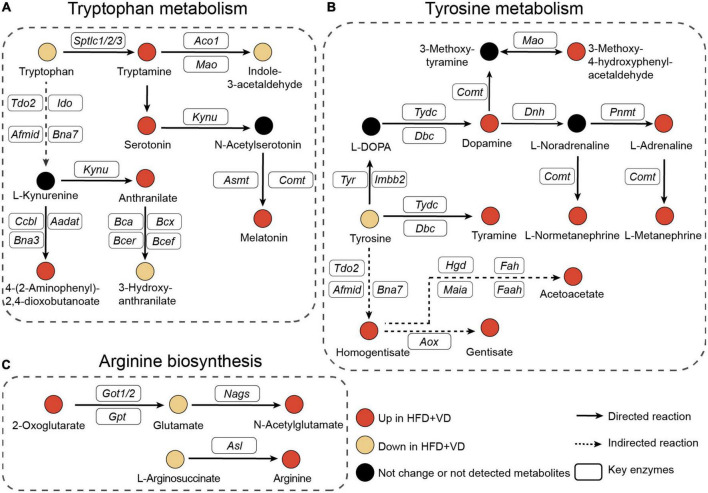
Pathway’s analysis. **(A–C)** Illustrative examples of metabolite changes in tryptophan metabolism, tyrosine metabolism, and arginine biosynthesis pathways.

Next, as illustrated in Figure 6A, the pathway analysis revealed that VD treatment increased the metabolizing ability of tryptophan, which may lead to the production of more tryptamine, serotonin, and melatonin, and the enhanced capability of tyrosine metabolism was also observed in the HFD + VD group (Figure 6B). Furthermore, VD intervention induced the N-acetylglutamate and arginine biosynthesis from glutamate and L-argininosuccinate, respectively (Figure 6C).

### Integrated analysis of the gut microbiota and metabolism

**FIGURE 7:**
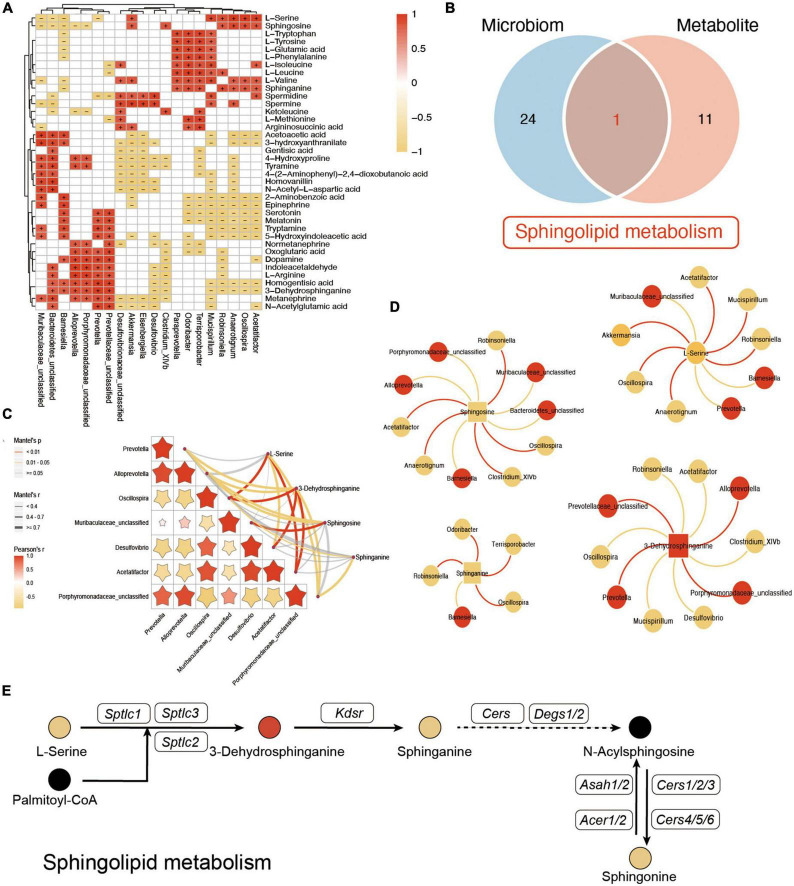
Integrated analysis between microbiome and metabolites. **(A)** The Spearman correlation analysis between the significantly changed microbiome and metabolites. + and – indicate the significant positive and negative association, respectively. **(B)** Venn diagram of significant differential KEGG pathways in functional analysis of gut microbiota and the metabolic pathway based on the metabolites. **(C,D)** Mantel’s analysis and interaction network plots of enriched metabolites in the shared pathways with microbes that participate in its metabolism. Red colors indicate significantly upregulated metabolites or microbes, while yellow colors indicate significantly downregulated metabolites or microbes. Red and yellow lines indicate the positive correlation and negative correlation, respectively. **(E)** Illustrative example of metabolite changes in the sphingolipid metabolism pathway.

To further explore the potential influence of VD in both gut microbiota and plasma metabolites, the Spearman analysis was performed to connect the changed metabolites and bacteria. As shown in Figure 7A, L-tryptophan, L-tyrosine, L-glutamic acid, L-phenylalanine, L-isoleucine, L-leucine, L-valine, and sphinganine, which were downregulated after VD treatment, were positively associated with *Clostridium_XlVb, Paraprevotella*, and *Mucispirillum*, which were lower in the HFD + VD group. *Prevotella, Prevotellaceae_unclassified*, and *Alloprevotella* were correlated with dopamine, L-arginine, indoleacetaldehyde, and 3-dehydrosphinganine. In addition, *Mucispirillum, Desulfovibrio*, and *Akkermansia* maintained a negative association with 4-hydroxyproline and tyramine. The enriched *Oscillospira, Robinsoniella*, and *Acetatifactor* in the HFD group were negatively correlated with upregulated 2-aminobenzoic acid, epinephrine, tryptamine, serotonin, and melatonin after VD treatment. It was noted that the integrated analysis of significantly changed pathways revealed that sphingolipid metabolism overlaps with microbiome and metabolite (Figure 7B). Furthermore, Mantel’s test and Spearman’s analysis were taken to associate the metabolites which belong to sphingolipid metabolism and altered gut bacterial (Figures 7C, D). Specifically, HFD + VD groups’ rats enriched in *Prevotella* and *Alloprevotella* were negatively correlated with *Acetatifactor, Oscillospira, and Desulfovibrio*, while maintaining a strong positive association with 3-dehydrosphinganine. Importantly, even though VD treatment induced the 3-dehydrosphinganine directly synthesis from L-serine and palmitoyl-CoA, the following metabolism to produce sphinganine and sphingosine was downregulated in the HFD + VD group (Figure 7E).

## Discussion

It has been well known that the high prevalence of NAFLD is becoming a key health concern globally (Draijer et al., 2019). More importantly, emerging evidence has demonstrated that gut microbiota is involved in NAFLD progression (Leung et al., 2022). VD, as an essential nutrient, benefits the bone growth. Additionally, low serum VD levels are at increased risk of multiple adverse health outcomes including obesity, incident diabetes, and autoimmune diseases (Barbachano et al., 2017). However, limited studies have been focused on the correlation between VD and NAFLD so far. Our previous study focuses on the effect of VD on hepatic injury, lipid accumulation, activation of the NLRP3 inflammasome, and pyroptosis (Zhang et al., 2021c). The dysbiosis of gut microbiota indicated the potential therapy against NAFLD. In this study, we further investigated the beneficial effects of VD on NAFLD by regulating the gut microbiota and metabolism. Our results indicate that the supplement of VD ameliorated the HFD-induced liver function and reversed the hepatic steatosis by modulating the gut microbiota.

The VD treatment significantly decreased the F/B ratio, the indicator of calorie absorption capability (Komaroff, 2017). The increased Chao1 index of gut microbiota in the HFD + VD group indicated that the VD treatment could restore the decreased species diversity induced by HFD. The *Bacteroidetes* phylum, usually decreased in patients with NAFLD, (Del Chierico et al., 2017) was reduced in the HFD group but could be reversed by VD supplement. At the genus level, the *Lactobacillus*, as documented to ameliorate the progression of NAFLD through the modulation of the gut microbiome in mice, (Jang et al., 2019; Lee et al., 2020) could also be induced after VD treatment. Similar results in rats showed that VD could restore HFD-induced gut microbiota dysbiosis by increasing the relative abundance of *Lactobacillus* and decreasing the relative abundance of *Acetatifactor*, *Oscillibacter*, and *Flavonifractor* (Zhang et al., 2021c). Furthermore, *Porphyromonadaceae* and *Ruminococcaceae*, which have been identified as butyrate-generating bacteria with health benefit effects, (Zhang et al., 2021a) were increased by VD supplement in HFD rats. Also, the *Prevotella*, negatively correlated with NAFLD severity, (Schwimmer et al., 2019) was increased after VD intervention. In addition, *Mucispirillum, Desulfovibrio*, and *Desulfovibrionaceae*, enriched in the high-fat/high-cholesterol-induced NAFLD mice, (Zhang et al., 2021b) were decreased after VD treatment. The increased level of *Acetobacter*, which could induce gut dysfunction, however, was decreased in the HFD + VD group. Further function analysis of gut microbiota indicates that the VD treatment significantly increased the linoleic acid metabolism capability of gut microbiota. Consistent with our findings, it has been reported that HFD could inhibit linoleic acid metabolism by altering the gut microbiota (Miyamoto et al., 2019). Taken together, our findings indicate that the VD could alleviate the NAFLD based on gut microbiota restoration.

The VD treatment reversed the metabolic dysbiosis as shown by metabolism analysis. Patients with metabolic syndrome could decrease the capacity of the microbiota to metabolize tryptophan into derivatives (Natividad et al., 2018; Wrzosek et al., 2021). However, we found that tryptophan metabolism was enhanced after VD treatment. Higher levels of tryptophan derivatives, such as tryptamine, serotonin, anthranilate, and melatonin, were observed in HFD + VD rats. Other reports have identified tryptamine as a metabolite that depends on the microbiota and is depleted under an HFD (Krishnan et al., 2018). Recently, research also revealed that serotonin and melatonin could regulate appetite and safeguard against fatty liver, respectively (Li et al., 2019). In addition, a previous study also revealed that tyrosine levels were positively associated with the presence of NAFLD (Gobeil et al., 2022). The lower level of tyrosine and a higher level of its derivatives imply that the VD treatment promotes the capability of tyrosine metabolism. Another study found that the bacteria in patients who received the weight-loss intervention had a higher capacity to produce tyramine from tyrosine (Li et al., 2021). The gut microbiota-derived dopamine could also regulate food seeking (Fernandes et al., 2020). Furthermore, an increase in adrenaline, normetanephrine, and changes in cecal microbiota, can induce the energy metabolism of fat tissue (Larabee et al., 2020). Other authors have proposed that *Akkermansia muciniphila* benefits metabolic syndrome by specific modulation of acetoacetate (Depommier et al., 2021). It has been documented that a higher level of glutamate increases the risk for obesity and metabolic syndrome and promotes hepatic gluconeogenesis (Andres-Hernando et al., 2021; Zhuang et al., 2021). However, the VD treatment not only inhibits the glutamate synthesis from 2-oxoglutarate but also promotes its metabolism to produce N-acetylglutamate. Dietary arginine could prevent intestinal inflammation in mice (Alexander et al., 2022). The arginine metabolism, as a potential immunomodulatory pathway, is mediated by *Bifidobacterium longum* and *A. muciniphila* (Strazar et al., 2021). However, the VD treatment promotes arginine synthesis from L-argininosuccinate. Thus, these results suggest that VD treatment may not only reverse the gut microbiota disorder but also restore metabolic dysbiosis.

Importantly, the integrated analysis of gut microbiota and metabolites also revealed a positive correlation between tryptophan, tyrosine, glutamic acid, phenylalanine, BCAAs, sphinganine, spermidine, and *Mucispirillum*, suggesting that the elevated level of these metabolites in HFD rats may be due to the enrichment of *Mucispirillum*. Increasing evidence has demonstrated that gut microbiota-derived sphingolipids could modulate hepatic metabolism (Le et al., 2022). Sphingolipids were reported to be generated by *Bacteroidetes*, the dominant phylum of the gut microbiome (Johnson et al., 2020). Our results showed that 3-dehydrosphinganine was positively correlated with *Prevotella* under the *Bacteroidetes* phylum. It has been reported that the pathway for bacterial sphingolipid synthesis is common in *Prevotella* (Heaver et al., 2022). Our results indicated that the enhancement of *Prevotella* abundance after VD treatment might promote the 3-dehydrosphinganine synthesis from serine. Sphingosine could regulate intestinal immune cells to protect the gut from infection; (Sun et al., 2022) however, sphingosine was also enriched in patients with NAFLD (Oh et al., 2020). In the present study, the positive correlation between sphingosine and *Oscillospira* suggests that the decline of *Oscillospira* after VD treatment may promote sphingosine synthesis. However, whether *Prevotella* and *Oscillospira* affect 3-dehydrosphinganine and sphingosine synthesis needs further study. This study has a few limitations. First, the role of vitamin D in obesity was different between men and women, and our study only uncovered the effects of VD on gut microbiota and metabolism in NAFLD without gender-dependent manners. A second limitation of this study is linked to the number of detected metabolites, which could be further improved by using high-resolution mass spectrometry, which accurately distinguishes isomer metabolites, especially lipids and their derivatives. Even though the aim of this study was to reveal the previously unknown VD supplement characteristics based on gut microbiota and metabolic profile to provide the clue for NAFLD prevention and treatment, it is a rather descriptive, and follow-up study on one of the hypotheses indicated by the omics analysis, such as fecal microbiota transplantation from vitamin D-supplemented rats to NAFLD rats or germ-free animal model validation, needs to be further studied.

## Conclusion

VD supplements alleviated the hepatic lipid accumulation induced by HFD. The present analysis of rats’ fecal bacteria and metabolites revealed the function of VD supplements in restoring the gut microbiota and metabolism dysbiosis. VD treatment induced the abundance of *Prevotella* which positively correlated with serotonin, melatonin, tryptamine, L-arginine, and 3-dehydrosphinganine. The reduction of *Mucispirillum* after VD supplement kept a positive correlation with plasma tryptophan, tyrosine, glutamic acid, phenylalanine, BCAAs, sphinganine, and spermidine, indicating that the VD could promote the tryptophan metabolism, tyrosine metabolism, and arginine biosynthesis *via* inhibiting the proliferation of *Mucispirillum*. In addition, VD treatment promotes sphingolipid metabolism in both gut microbiota function and metabolite pathway. This comprehensive integrated microbiota and metabolomic analysis provide insights into the relationship between the VD, fecal microbiome, and the deregulation of metabolism in process of HFD-induced NAFLD, suggesting that VD supplement could be a potential intervention used for NAFLD treatment by targeting the specific microbiota.

## Data availability statement

The data presented in the study are deposited in the NCBI repository, accession number PRJNA928563.

## Ethics statement

This animal study was reviewed and approved by the Ethics Committee of China Medical University (No. 2018162).

## Author contributions

X-LZ, JiaY, LC, S-SZ, SJ, and NA designed and performed the experiments. LC conducted the analyses and wrote the manuscript. X-LZ measured the fecal bacteria and metabolites. H-XL and JD conceived and supervised the study. All authors read and approved the final version of the manuscript.

## Footnotes

1
http://www.metaboanalyst.ca
