# Supplementary material for: Vitamin D alleviates non-alcoholic fatty liver disease via restoring gut microbiota and metabolism
Source: Front Microbiol. 2023 Feb 2;14:1117644. doi: 10.3389/fmicb.2023.1117644 (PMC9932697; doi:10.3389/fmicb.2023.1117644)
Supplement: Supplementary file 1 [file Table_1.docx]

**Table S1. Significantly changed of the plasma metabolome (HFD+VD vs HFD).**

| Name | Log2FC | KEGG ID |
| --- | --- | --- |
| Epinephrine | 21.92 | C00788 |
| 9,10-DHOME | 21.82 | C14828 |
| 9,10-EOT | 21.64 | C16324 |
| Calcitriol | 21.32 | C01673 |
| (S)-2-amino-6-oxohexanoate | 20.29 | C04076 |
| Serotonin | 20.28 | C00780 |
| trans-Cinnamate | 20.06 | C00423 |
| Tyrosol | 19.69 | C06044 |
| 2-Keto-6-acetamidocaproate | 17.90 | C05548 |
| 4-Hydroxyproline | 16.89 | C01157 |
| 2-Aminobenzoic acid | 16.36 | C00108 |
| Tyramine | 15.86 | C00483 |
| Melatonin | 13.57 | C01598 |
| 4-(2-Aminophenyl)-2,4-dioxobutanoic acid | 6.96 | C01252 |
| Xanthurenic acid | 6.20 | C02470 |
| 2-Dehydropantoate | 6.18 | C00966 |
| Gentisic acid | 5.75 | C00628 |
| Tryptamine | 5.30 | C00398 |
| 1-Naphthylamine | 5.10 | C14790 |
| Jasmonic acid | 4.88 | C08491 |
| Lumichrome | 4.68 | C01727 |
| Acetaminophen | 4.41 | C06804 |
| Pyridoxine | 4.16 | C00314 |
| Dehydroepiandrosterone | 3.99 | C01227 |
| 3-hydroxyanthranilate | 3.67 | C00632 |
| 3-Hydroxyphenylacetic acid | 3.57 | C05593 |
| Adenine | 3.28 | C00147 |
| 2-Deoxyadenosine | 3.04 | C00559 |
| Vitamin D3 | 3.00 | C05443 |
| Glucosamine | 2.96 | C00329 |
| Metanephrine | 2.80 | C05588 |
| D-Mannose | 2.76 | C00159 |
| 3-(2-Hydroxyphenyl)propanoic acid | 2.72 | C01198 |
| Salicylic acid | 2.71 | C00805 |
| 3-Oxalomalate | 2.64 | C01990 |
| Nicotinamide riboside | 2.58 | C03150 |
| Cytosine | 2.26 | C00380 |
| Glutaric acid | 2.22 | C00489 |
| N-Acetyl-L-glutamate 5-semialdehyde | 2.19 | C01250 |
| Guanine | 2.12 | C00242 |
| N-Acetyl-L-aspartic acid | 2.07 | C01042 |
| Homogentisic acid | 2.06 | C00544 |
| 6-Hydroxynicotinic acid | 2.03 | C01020 |
| Nicotinic acid | 2.02 | C00253 |
| L-Lactic acid | 2.00 | C00186 |
| Acetoacetic acid | 1.96 | C00164 |
| L-Arginine | 1.87 | C00062 |
| D-Phenyllactic acid | 1.71 | C05607 |
| Normetanephrine | 1.70 | C05589 |
| N-Acetylglutamic acid | 1.69 | C00624 |
| 3-Dehydrosphinganine | 1.59 | C02934 |
| Pimelic acid | 1.52 | C02656 |
| Homovanillin | 1.48 | C05581 |
| 11-Dehydro-thromboxane B2 | 1.45 | C05964 |
| L-2-Hydroxyglutaric acid | 1.42 | C03196 |
| Uracil | 1.40 | C00106 |
| N-Acetylneuraminate | 1.37 | C00270 |
| Alpha-Tocotrienol | 1.37 | C14153 |
| 3-Hydroxybenzoic acid | 1.32 | C00587 |
| 2-Hydroxy-3-oxoadipate | 1.30 | C03217 |
| Dopamine | 1.26 | C03758 |
| Mesaconate | 1.26 | C01732 |
| O-Acetyl-L-homoserine | 1.24 | C01077 |
| 13-L-Hydroperoxylinoleic acid | 1.20 | C04717 |
| N-Acetylmuramate | 1.17 | C02713 |
| Oxoglutaric acid | 1.15 | C00026 |
| D-Ribose | 1.09 | C00121 |
| Glycerophosphocholine | 1.02 | C00670 |
| D-Cathinone | 0.97 | C08301 |
| Aspartame | 0.96 | C11045 |
| 9-cis-Retinal | 0.95 | C16681 |
| Xanthine | 0.83 | C00385 |
| N-a-Acetylcitrulline | 0.73 | C15532 |
| Subaphylline | 0.62 | C10497 |
| Indoleacetaldehyde | 0.59 | C00637 |
| 5-Hydroxyindoleacetic acid | 0.12 | C05635 |
| 1-Aminocyclopropanecarboxylic acid | -0.64 | C01234 |
| Fructose 1,6-bisphosphate | -0.75 | C00354 |
| L-2,4-diaminobutyric acid | -0.86 | C03283 |
| Imidazolepropionic acid | -0.97 | C20522 |
| Ketoleucine | -1.02 | C00233 |
| Urocanic acid | -1.02 | C00785 |
| L-Valine | -1.07 | C00183 |
| 4-Hydroxycinnamic acid | -1.10 | C00811 |
| Argininosuccinic acid | -1.11 | C03406 |
| Pyridoxamine 5-phosphate | -1.12 | C00647 |
| 21-Hydroxypregnenolone | -1.20 | C05485 |
| 2-Phenylacetamide | -1.23 | C02505 |
| L-Phenylalanine | -1.24 | C00079 |
| L-Tyrosine | -1.25 | C00082 |
| 5,6-Dihydro-5-fluorouracil | -1.31 | C16630 |
| Mannitol | -1.34 | C00392 |
| L-Methionine | -1.37 | C00073 |
| L-Serine | -1.37 | C00065 |
| L-Arabinonate | -1.38 | C00545 |
| Prostaglandin F2a | -1.46 | C00639 |
| 2-Hydroxybutyric acid | -1.50 | C05984 |
| Nopaline | -1.59 | C01682 |
| L-Glutamic acid | -1.69 | C00025 |
| L-Methionine S-oxide | -1.75 | C02989 |
| Sphinganine | -1.76 | C00836 |
| Diethanolamine | -1.81 | C06772 |
| D-1-Piperideine-2-carboxylic acid | -1.86 | C04092 |
| D-Fructose | -1.87 | C00795 |
| 4-Hydroxyphenylacetylglutamic acid | -1.88 | C05595 |
| Spermidine | -2.00 | C00315 |
| 6-Phosphogluconic acid | -2.01 | C00345 |
| p-Octopamine | -2.04 | C04227 |
| 6-Ketoprostaglandin E1 | -2.18 | C05962 |
| Piperidine | -2.19 | C01746 |
| L-Leucine | -2.21 | C00123 |
| Desmosterol | -2.35 | C01802 |
| L-Tryptophan | -2.35 | C00078 |
| Saccharopine | -2.44 | C00449 |
| 9-cis-Retinoic acid | -2.67 | C15493 |
| Sphingosine | -2.76 | C00319 |
| Spermine | -2.84 | C00750 |
| L-Isoleucine | -2.88 | C00407 |
| Taurocholic acid | -2.97 | C05122 |
| Berberine | -3.00 | C00757 |
| 1-Hexadecanol | -3.15 | C00823 |
| Stearic acid | -3.16 | C01530 |
| 21-Deoxycortisol | -3.61 | C05497 |
| 7-Dehydrocholesterol | -3.81 | C01164 |
| Cortisone | -3.87 | C00762 |
| 20a,22b-Dihydroxycholesterol | -4.81 | C05501 |
| Isovaleric acid | -6.28 | C08262 |
